# Metabolomic Analysis of Patients with Coronary Artery Disease and Acute Myocardial Infarction by Using Nuclear Magnetic Resonance Spectroscopy

**DOI:** 10.1007/s12265-026-10866-5

**Published:** 2026-11-03

**Authors:** Nikolaos Pagonas, Werner Dammermann, Gerasimos Siasos, Sawa Kostin, Benjamin Sasko, Philipp Sotiriou, Selina Strathmeyer, Steffen Heelemann

**Affiliations:** 1 Department of Internal Medicine and Cardiology, Medical School Theodor Fontane, University Hospital Ruppin-Brandenburg, 16816 Neuruppin, Germany; 2 Department of Gastroenterology, Diabetology and Hepatology, Center for Internal Medicine II, University Hospital Brandenburg an der Havel, Medical School Theodor Fontane, 14770 Brandenburg an der Havel, Germany; 3 Center for Translational Medicine, Brandenburg Medical School Theodor Fontane, 14770 Brandenburg an der Havel, Germany; 4 Faculty of Health Sciences Brandenburg, Brandenburg Medical School Theodor Fontane, 16816 Neuruppin, Germany; 5 3rd Department of Cardiology, Medical School, “Sotiria’’ Chest Disease Hospital, National and Kapodistrian University of Athens, 11527 Athens, Greece; 6 Medical Department II, Marien Hospital Herne, Ruhr-University of Bochum, 44625 Herne, Germany; 7 lifespin GmbH, 93053 Am BioPark 13, Regensburg, Germany

**Keywords:** Acute myocardial infarction, Coronary artery disease, Nuclear magnetic resonance, Metabolomics

## Abstract

This cross-sectional exploratory study investigates the potential of metabolomics to differentiate between stable coronary artery disease (CAD) and acute myocardial infarction (AMI) through Nuclear Magnetic Resonance (NMR) spectroscopic analysis. The study identifies distinct metabolic profiles for each condition, revealing significant variations in metabolite levels, particularly lactic acid, which is a key metabolic product of anaerobic glycolysis, emerges as a prominent marker in AMI patients, reflecting acute ischemic stress and providing insight into adverse outcomes associated with high lactate levels. Another metabolite found in the present study was hypoxanthine, which is key biomarker for myocardial ischemia. These findings underscore the potential of metabolomics in combination with pathway analysis in distinguishing between CAD and AMI and highlight the metabolic dysregulation that occurs in these diseases. Our results suggest that these metabolites could serve as potential biomarkers, paving the way for personalized prognostic and diagnostic strategies. Further large-scale studies are necessary to validate these findings and incorporate them into clinical practice.

**Supplementary Information:**

The online version contains supplementary material available at https://doi.org/10.1007/s12265-026-10866-5.

## Introduction

Coronary artery disease (CAD) and acute myocardial infarction (AMI) are pivotal concerns in cardiovascular pathology. CAD, as the predominant manifestation of heart disease, frequently leads to AMI, a critical condition characterized by abrupt occlusion of coronary arteries. These two manifestations exhibit distinct metabolic profiles due to their different underlying pathophysiological mechanisms. Metabolomics, which focuses on analyzing small molecules involved in metabolism, represents a promising approach for identifying novel biomarkers. Through comprehensive analysis of metabolites in blood samples, this field offers opportunities to both enhance our understanding of disease mechanisms and uncover potential therapeutic targets. Recent literature emphasizes the necessity of elucidating the divergent effects of CAD and AMI on patient prognoses, thereby underscoring the imperative for targeted comparative studies. For this purpose, both clinical and metabolomic differences between CAD and AMI patients and healthy controls must be investigated to identify actionable therapeutic targets. Employing this comparative methodology not only facilitates improved management strategies for CAD and AMI but also propels advancements in personalized medicine, potentially optimizing outcomes through patient-specific interventions.

Nuclear magnetic resonance (NMR) technology plays a pivotal role in advancing our understanding of coronary artery disease (CAD) by providing detailed insights into the molecular mechanisms underlying the disease. NMR enables the quantification of small molecules and lipoproteins in blood [1]. Thus, it facilitates the identification of early biomarkers and pathological changes in CAD and AMI [2]. This technology’s ability to non-invasively assess myocardial composition and vascular integrity makes it invaluable in diagnosing CAD at its nascent stages and evaluating the effectiveness of various treatments [3]. Integrating NMR into current CAD management protocols can revolutionize how clinicians assess risk, tailor patient-specific therapies, and monitor disease progression, potentially leading to more accurate prognoses and improved outcomes [4].

## Methods

### Study Design

The study was conducted at the University Hospital of Ruppin-Brandenburg. We enrolled two independent cohorts. The first cohort consisted of patients admitted for elective coronary angiography between 2018 and 2019. Inclusion criteria were known or suspected diagnosis of CAD and a study independent indication for elective coronary angiography. Exclusion criteria were known cancer disease, acute infectious disease, known rheumatic disease and age < 18 years. The second cohort consisted of patients with ACS, admitted into the emergency department between 2019 and 2021. All patients with ACS underwent urgent coronary angiography. Patients with ACS met the same exclusion criteria as the elective patients. Patients tested positive for COVID-19 were excluded from analysis. Based on the findings of angiography, those patients were assigned to the CAD group who had an at least 50% stenosis in the diameter of a coronary artery. Patients with acute myocardial infarction and confirmed epicardial coronary disease were recruited in the AMI group. Written informed consent was obtained from all participants. The study was approved by the local ethics committees of the medical association of Brandenburg (Nr. AS69bB/2016) in accordance with the Declaration of Helsinki.

### Routine Laboratory Assessment

Blood sampling was performed after overnight fasting before any invasive diagnostics in the CAD group or within 24 h after hospital admission in the AMI group (mostly after coronary angiography). The serum was prepared from blood samples and was cryopreserved (-80 °C). Laboratory parameters were obtained by standard clinical assays in the central laboratory unit of the University Hospital Ruppin-Brandenburg, Germany. High-sensitivity C-reactive protein (hs-CRP) was measured using the Roche Tina-Quant CRP (Latex) kit (Roche Diagnostics, Basel, Switzerland).

### NMR Measurement and Metabolomic Profiling

NMR measurements were conducted at lifespin GmbH. After thawing samples (approx. 3 h at room temperature), 350 µl of serum were taken and mixed with 350 µl of aqueous buffer. The buffer consists of H_2_O p.A., 0.1 g/l NaN_3_, 0.067 mol/l Na_2_HPO_4_, 0.033 mol/l NaH_2_PO_4_ (pH: 7.15 ± 0.05), 5% D_2_O as field-lock substance and an internal standard (6 mM pyrazine) for quantification. From this mixture, 600 µl were transferred into a 5 mm Bruker NMR tube and sealed with barcode-labelled lids. The final NMR samples were stored after preparation at refrigerator temperature until measurement, max. 24 h. NMR measurement was performed with the following parameters: Spectrometer: Bruker AVANCE NEO 600 MHz, measuring method: 1D 1 H noesygppr1d_d20, NS = 16, T=310 K, measuring time per sample: 6.5 min. All measured spectra passed the quality control routine and were released for data analysis. The spectra obtained were Fourier-transformed using TopSpin software (version 4.2.0, Bruker Biospin, Germany) and all spectra were automatically phased and subjected to baseline correction. Subsequently, the spectra were analyzed using the proprietary lifespin Profiler software (version 1.4_Blood) and Lipoprotein Profiler software (version 1.2.3_A) and a quantitative metabolite list was generated. Metabolite concentrations below the limit of detection were set to zero.

### Statistical Analysis

Statistical analyses were performed using R, version 4.0.2 and 4.4.1. Continuous variables are presented as mean and range (minimum–maximum), or as median and interquartile range (IQR, 25th–75th percentile), depending on the distribution. Categorical variables are presented as absolute and relative frequencies.

For Table 1, group differences were assessed using the Wilcoxon rank-sum test or chi-square test, as appropriate. In Table 2, metabolite-wise group differences were tested using the Wilcoxon rank-sum test for unpaired, non-normally distributed data. Resulting p-values were adjusted for multiple testing across all 76 metabolites using the Benjamini-Hochberg false discovery rate procedure (FDR). Adjusted p-values were denoted as follows: *p* ≤ 0.001 (***), *p* ≤ 0.01 (**), *p* ≤ 0.05 (*) and *p* > 0.05 (n.s., not significant). Due to potential zero inflation, the Wilcoxon rank-sum test was interpreted as a test for differences in the overall rank distribution between groups rather than strictly as a test for median differences.

**Table 1:** Demographic overview of patient cohort

Feature	CAD	AMI	*p*-value
Number	82	52	
Age, years^+^	72 (40–92)	71 (42–92)	0.7028
Male, no. (%)	60 (73.2)	36 (69.2)	0.7669
Hypertension, no. (%)	79 (96.3)	42 (80.8)	0.01524*
Diabetes, no. (%)	26 (31.7)	16 (30.8)	1
Current smoking, no. (%)	15 (18.3)	11 (21.2)	0.6305
Adipositas, no. (%)	23 (28)	20 (38.5)	0.2853
Kidney insufficiency, no. (%)	32 (39)	17 (32.7)	0.5771
Hyperlipidaemia, no. (%)	81 (98.8)	46 (88.5)	0.1312
Statins intake, no. (%)	69 (84.1)	28 (53.8)	0.001348**
BMI, kg/m^2#^	27.1 (25.1; 30.81)	28.1 (26.01; 31.54)	0.1694
HDL, mg/dl^#^	47.75 (39.72; 61.57)	43.3 (35.76–51.61)	0.02602*

Information in () is either percentage, or mean (min-max), or median (25% to 75% IQR), the latter two indicated by + and # respectively. BMI = body mass index, HDL = high-density lipoprotein, no. = number. Statistical significance was assessed with Wilcoxon Rank Sum Test or Chi-Square Test. Significance asteriks: *** (*p* < 0.001), ** (*p* < 0.01), * (*p* < 0.05)

**Table 2:** Statistical Significance for CAD (*n* = 82) vs. AMI (*n* = 52)

Metabolite	*p*-value (corr.)	*-notation (corr.)	log2 fold change (95% confidence intervals)	Cliff’s Delta
Lactic acid	9.96e-13	***	-1.533 (-1.825; -1.215)	-0.792
Hypoxanthine	1.27e-11	***	-3.083 (-4.331; -2.257)	-0.642
Formic acid	2.91e-11	***	-0.982 (-1.238; -0.737)	-0.731
Glutamic acid	2.96e-11	***	-0.971 (-1.226; -0.727)	-0.723
Phenylalanine	2.96e-11	***	-0.831 (-1.013; -0.642)	-0.726
Glycine	1.34e-09	***	-0.458 (-0.602; -0.313)	-0.664
Glutamine	1.70e-09	***	0.331 (0.239; 0.422)	0.658
Isoleucine	1.96e-08	***	-0.479 (-0.617; -0.348)	-0.616
Isopropanol	3.07e-08	***	-4.033 (-5.671; -1.184)	-0.583
Aspartic acid	5.37e-08	***	-3.61 (-22.146; -2.209)	-0.411
Leucine	5.37e-08	***	-0.393 (-0.51; -0.272)	-0.593
Succinic acid	2.68e-07	***	-1.787 (-3.243; -0.245)	-0.563
Taurine	3.75e-07	***	-0.676 (-0.965; -0.359)	-0.556
Serine	3.93e-07	***	-0.461 (-0.618; -0.308)	-0.553
Methanol	1.54e-06	***	-0.869 (-1.172; -0.534)	-0.526
Valine	4.96e-06	***	-0.297 (-0.397; -0.197)	-0.502
Glycerol	5.24e-06	***	-1.587 (-2.172; -0.937)	-0.5
Acetic acid	5.81e-06	***	-0.757 (-1.174; -0.38)	-0.496
Ornithine	1.36e-05	***	-0.497 (-0.694; -0.314)	-0.477
Asparagine	2.82e-05	***	-0.864 (-1.216; -0.546)	-0.456
2-Aminobutyric acid	5.47e-05	***	-0.981 (-1.52; -0.527)	-0.427
Creatine	2.48e-04	***	-0.815 (-1.263; -0.353)	-0.408
Choline	7.37e-04	***	-0.453 (-0.724; -0.18)	-0.379
2-Methyl-1,3-propanediol	7.68e-04	***	-2.473 (-4.11; -1.307)	-0.243
Betaine	1.15e-03	**	-1.608 (-3.452; -0.354)	-0.24
Lysine	1.15e-03	**	-0.243 (-0.369; -0.116)	-0.364
Ethanolamine	1.23e-03	**	-2.913 (-19.839; -1.156)	-0.186
Acetone	1.26e-03	**	-0.883 (-1.338; -0.408)	-0.36
Glucose	2.54e-03	**	0.318 (0.036; 0.64)	0.339
3-Hydroxybutyric acid	3.18e-03	**	-1.173 (-1.994; -0.381)	-0.332
Proline	7.09e-03	**	-0.303 (-0.475; -0.142)	-0.306
Ascorbic acid	2.07e-02	*	2.015 (0.335; 19.853)	0.164
Histidine	2.65e-02	*	-0.148 (-0.258; -0.041)	-0.26
Citric acid	3.08e-02	*	-0.409 (-0.767; -0.036)	-0.253
2,3-Butanediol	3.13e-02	*	-1.967 (-17.898; 0.049)	-0.109
Pyruvic acid	3.25e-02	*	-0.801 (-1.454; 0.018)	0.249
Mannose	3.48e-02	*	0.316 (-0.023; 0.688)	0.244
Dimethylmalonic acid	3.48e-02	*	1.87 (0.095; 16.196)	0.142
Dimethylamine	3.55e-02	*	-0.813 (-1.579; -0.065)	-0.201
Mannitol	4.43e-02	*	-3.546 (-23.717; -0.214)	-0.084

Group 1 CAD (*n* = 82), Group 2 AMI (*n* = 52). Statistical significance was determined using the Wilcoxon rank-sum test. After FDR-correction across all metabolites, the adjusted p-values were translated into * notation as follows: ***p ≤ 0.001, **p ≤ 0.01, *p ≤ 0.05, n.s. = not significant. Log2 fold change is mean-based, and pseudo count was set to 1.00e-08 for all metabolites. Concentration given in mmol/l (small metabolites) or mg/dl (lipoprotein parameters)

Effect size and direction was assessed using mean-based log2 fold change (i.e. log2 ratio of arithmetic means) with a fixed pseudocount of 1.00e-8, and Cliff’s delta. Positive values for log2 fold change and Cliff’s delta indicate higher values in the first group. Percentile-based 95% confidence intervals for log2 fold changes were estimated using non-parametric bootstrap resampling within groups (2000 iterations).

Partial least squares discriminant analysis (PLS-DA) was applied as an exploratory supervised multivariate analysis to assess class-related metabolomic differentiation between groups. Prior to model fitting, variables with near-zero variance were removed (caret package version 6.0.86, nearZeroVar function) and the data were mean-centered and scaled to unit variance. Model performance was assessed via cumulative R2X, R2Y and Q2 values, with Q2 values estimated by the default 7-segment cross-validation in ropls::opls(), version 1.20.0. Permutation testing was performed (500 permutations of class labels) and the permutation-derived p-values were reported as pR2Y and pQ2. PLS-DA score plot displays samples on the first two PLS components with 90% data ellipses per group. Labelled variables in the loading plot were selected based on loading strength and VIP (variable importance in projection) selection stability to determine their contribution to the supervised PLS-DA model.

To evaluate the PLS-DA model regarding robustness and potential overfitting, first repeated stratified 5-fold cross-validation was performed to test whether class separation was stable across many train/test splits. For this, accuracy and balanced accuracy were reported across repeated splits. Also, a permutation-based null distribution of repeated cross-validated balanced accuracy was performed to assess the performance of models with randomized class labels. Finally, via a subsampling-based VIP stability analysis, robustness was evaluated: by fitting the PLS-DA model on stratified 80% subsamples, the variables’ frequency among the top VIP-ranked variables was recorded.

A random forest model was trained (randomForest package) to distinguish between the metabolomic profiles of the analysed groups. Model performance was assessed using repeated stratified 10-fold cross-validation with 5 repeats. Within each training fold, near-zero variance predictors were removed, and then highly correlated predictors were filtered (Spearman correlation and a cut-off of 0.99). To avoid information leakage, these preprocessing steps were learned only from the training data and then applied to the corresponding test fold. Random forest models were fitted with 1000 trees, and class probabilities were predicted for the corresponding test subset. ROC curves and AUC values were used for performance evaluation. Fold-wise AUC values were summarized via mean and standard deviation. Additionally, a pooled cross-validated ROC curve and pooled AUC were calculated from all held-out predictions. For exploratory variable ranking, a final random forest model was fitted on the full pre-processed dataset, and variable importance was assessed using mean decrease in Gini impurity. To evaluate the internal stability of the predictor ranking, variable importance was recorded for each random forest model and the appearance frequency of the individual variables among the top 20 predictors (ranked by MeanDecreaseGini) across the cross-validated models was assessed.

No independent external validation cohort was available, and hence PLS-DA results and random forest results were considered as internally assessed and exploratory.

Identified, significantly altered metabolites were subjected to a pathway enrichment analysis using the FELLA R package, version 1.24.0, with the human KEGG database. The background list for this analysis comprised 64 metabolites, which were mapped to KEGG IDs. 12 detectable metabolites were excluded as no valid KEGG ID was available. The pathway analysis was based on the FELLA PageRank method using normality approximation, and results were restricted to pathways. Resulting pathways were ranked by the raw FELLA PageRank p-score, with smaller scores indicating stronger PageRank-based prioritization of the pathway.

## Results

### Baseline Characteristics

The participants’ characteristics are presented in Table 1. The study population included both male and female subjects, with males predominating significantly in both groups (73.2% in CAD and 69.2% in AMI. The average age of participants with CAD was 72 years, while those with AMI had an average age of 71 years A very high prevalence of pre-existing hypertension was noted in both groups; the CAD group has 96.3% while the AMI Group has 80.8% (*p* < 0.05). A statistically significant difference was observed in the use of statins, with 84.1% of participants in the CAD group taking statins compared to only 53.8% in the AMI group (*p* = 0.001). This is not uncommon, since many patients experience the first manifestation of CAD as an AMI. Furthermore, HDL was higher in the CAD group compared to AMI patients (*p* < 0.05).

### Statistical Analysis of Metabolomic Data

**Fig. 1:**
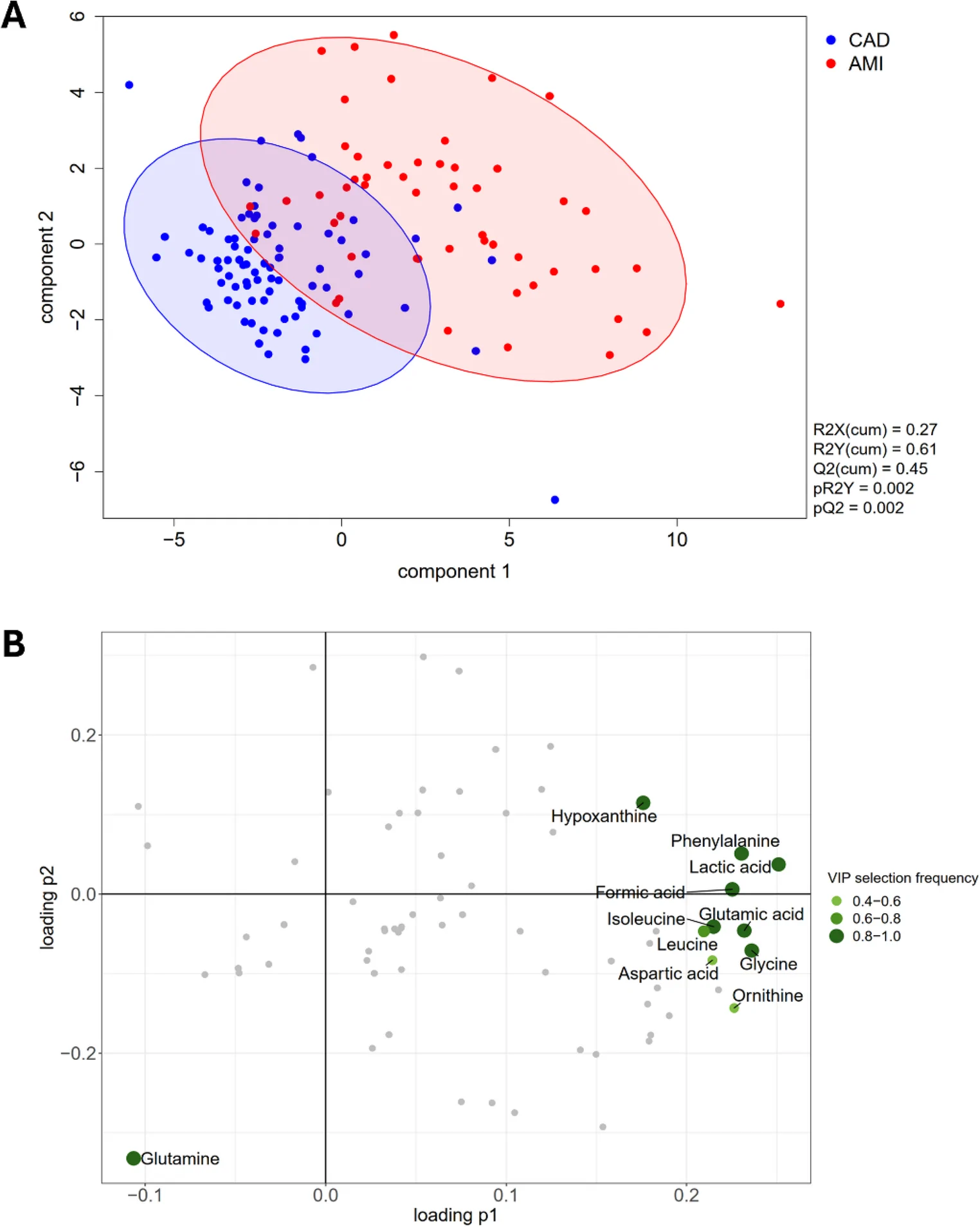
Supervised multivariate analysis by Partial Least-Squares Discriminant Analysis (PLS-DA). Score plot (**A**) and loading plot (**B**) for CAD vs AMI. Figure A shows the score plot from an exploratory PLS-DA for the comparison of CAD (depicted in blue) and AMI (depicted in red), plotted based on the first two components. Colored ellipses represent 90% data ellipses, and colored points represent individual samples. Cumulative R2X, R2Y and Q2 values are depicted besides the plot together with permutation-derived p-values pR2Y and pQ2 (500 class label permutations). Figure B depicts the loading plot based on the first two components. Each point represents one metabolite. Variables depicted in green and labelled by name were selected based on both visual contribution to the loading structure and stability in the subsampling based VIP (Variable Importance in Projection) analysis. Variables further from the origin contribute more to the loading structure and the point size indicates VIP selection frequency (the bigger, the more often selected)

As shown in Table 2, numerous biomarkers have been investigated and differed significantly between CAD and AMI. In this study, lactic acid (*p* = 9.96e-13) demonstrated the strongest statistical difference between CAD and AMI, followed closely by hypoxanthine (*p* = 1.27e-11), formic acid (*p* = 2.91e-11), glutamic acid (*p* = 2.96e-11) and phenylalanine (*p* = 2.96e-11). Statistically significant and of high biological relevance is also aspartic acid (*p* = 5.37e-08). Applying a PLS-DA for supervised multivariate analysis revealed separable clustering of the two indication groups (Fig. 1A) with good performance parameters of the exploratory model (R2X(cum) 0.27, R2Y(cum) 0.61, Q2(cum) 0.45). Internal robustness was positively assessed by repeated stratified cross-validation, a permutation-based null distribution of repeated cross-validated balanced accuracy, and subsampling-based VIP stability analysis (Fig S1). The variables highlighted in the respective loading plot(Fig. 1B) as contributing strongly to the group separation point toward similar changes in metabolites as presented in Table 2.

**Fig. 2:**
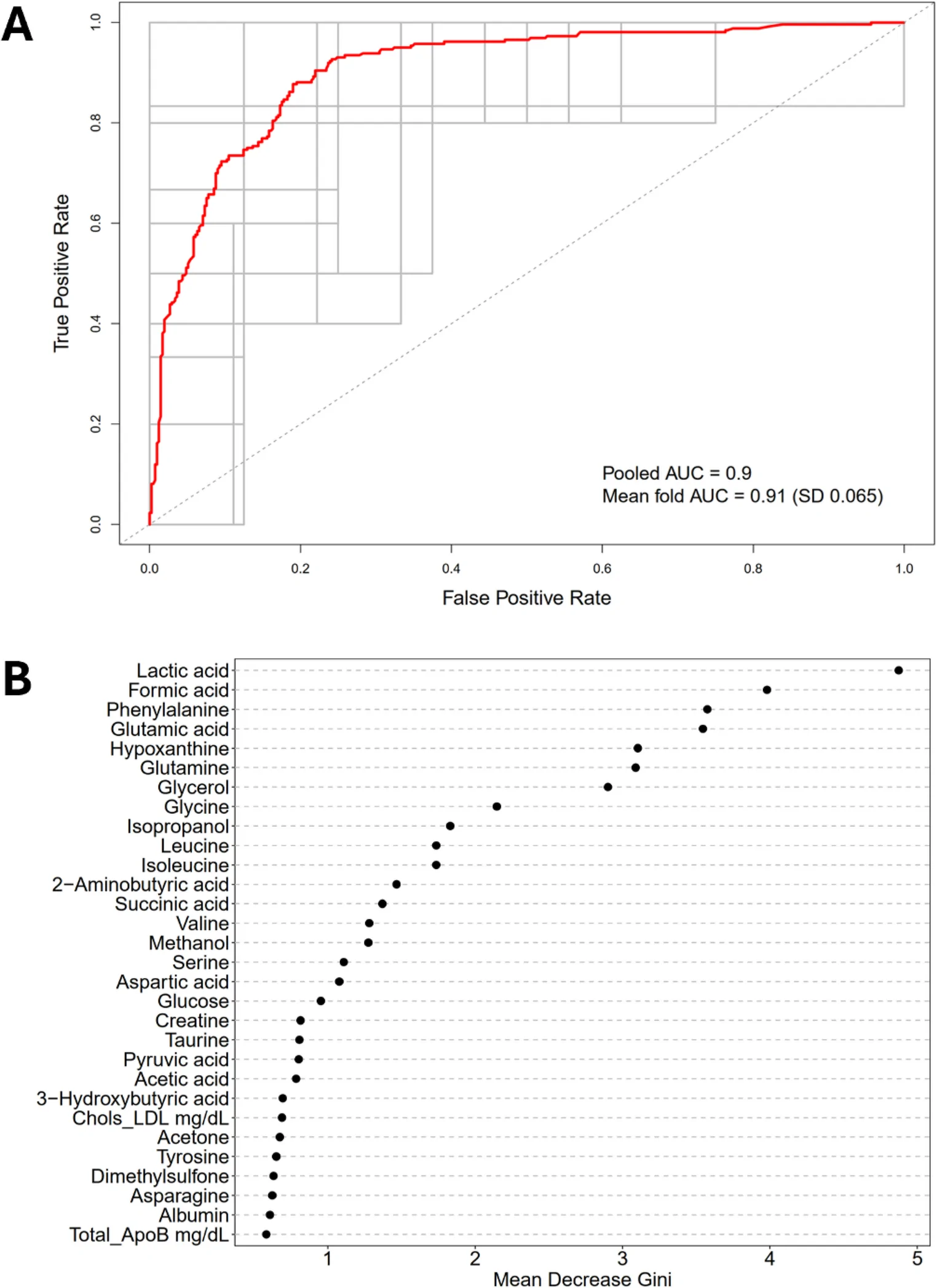
Cross-validated receiver operating characteristics (ROC) curves for the random forest classifier (cross-validated, 10-fold, 5 times) (**A**) and exploratory random forest variable importance based on mean decrease in Gini impurity (**B**). In Figure A, the grey lines show the empirical ROC curves from the individual cross-validation folds. The red line shows the pooled ROC curve calculated from all the held-out predictions (i.e. test datasets). Pooled area under the curve (AUC) value was calculated from the same held-out predictions. Descriptive summary given via mean fold-wise AUC and standard deviation (SD). Fold-wise AUCs are not independent due to repeated cross-validation. Figure B shows the top 30 predictors ranked by mean decrease in Gini impurity from the final random forest model (fitted on full preprocessed dataset). Higher values indicate a greater contribution of the predictor to reducing node impurity in the classification trees. Variable importance used for an exploratory ranking of metabolite contributions.

Training a random forest model to distinguish between AMI and CAD gave a high (pooled) AUC value of 0.9 (Fig. 2A), and the metabolites with the strongest Gini impurity index (Fig. 2B) were again in line with the metabolites detected in Table 2. To confirm that this variable ranking was not based on one single full-data model, the appearance frequency of the predictors for each cross-validated model based on MeanDecreaseGini was recorded and the top 20 predictors listed (Fig S2). Several predictors were repeatedly ranked among the most important variables and correspond well to the results from Table 2.

The changes in metabolites identified in the CAD and AMI groups by statistical significance were further investigated using the Kyoto Encyclopedia of Genes and Genomes (KEGG) database. The results are shown in Table 3.

**Table 3:** KEGG pathways based on FELLA PageRank analysis, CAD vs. AMI

KEGG ID	KEGG name	raw PageRank *p*-score
hsa05230	Central carbon metabolism in cancer	2.01e-26
hsa02010	ABC transporters	2.67e-26
hsa04978	Mineral absorption	2.71e-18
hsa01230	Biosynthesis of amino acids	2.03e-11
hsa00970	Aminoacyl-tRNA biosynthesis	4.19e-11
hsa04974	Protein digestion and absorption	8.81e-10
hsa00250	Alanine, aspartate and glutamate metabolism	1.57e-09
hsa00290	Valine, leucine and isoleucine biosynthesis	1.67e-09
hsa00650	Butanoate metabolism	5.61e-07
hsa04024	cAMP signaling pathway	2.03e-05
hsa00051	Fructose and mannose metabolism	0.0002

The table shows the KEGG pathways selected based on the statistically significant metabolites assessed for CAD vs. AMI. Pathways are ranked by the raw FELLA PageRank p-score, with smaller score indicating stronger PageRank-based prioritization of the pathway. Background for the analysis were all detectable metabolites of the present analysis which had valid KEGG compound annotations (no ID available for: 2-Methyl-1,3-propanediol, Albumin, Dimethylmalonic acid, Total Protein, Total CE, Total FC, Total PL, Total ApoA1, Total ApoA2, Total ApoB, Chols_HDL, Chols_LDL)

## Discussion

In this cross-sectional exploratory study, we examined the potential of metabolomics to distinguish between stable CAD and AMI through NMR spectroscopic analysis. The results revealed significant differences in metabolic profiles between these two conditions. Specifically, we identified a distinctive panel of metabolites, especially lactic acid, which showed particularly notable differences between the CAD and AMI patient groups.

The major findings of our work are based on the direct metabolomic comparison between AMI and stable CAD patients. By elucidating the distinct metabolic profiles differentiating these two conditions, our study provides new insights into their underlying pathophysiology. This comparative methodology is essential for improving risk stratification and offering patient-specific management strategies in line with advancements in personalized cardiology care.

In the current study, we find a strong signal for lactic acid in patients with AMI, reflecting the fact that patients with AMI suffer acutely from a reduced oxygen supply at least to one coronary artery. These results, which are in accordance with the above studies, may imply an adverse outcome in patients with higher lactic acid levels but this was not investigated in the current study population.

Lactic acid, predominantly produced through anaerobic glycolysis, has emerged as a key metabolic signature in various cardiovascular diseases. As a metabolic product of glycolysis, lactate plays a central role in cardiovascular metabolism, serving as an important energy substrate for cardiomyocytes under both aerobic and anaerobic conditions [5]. At rest, the heart derives 60–90% of its ATP from β-oxidation of fatty acids and 10–40% from pyruvate. Under physiological stress, lactate becomes the primary energy substrate. During physical exertion, lactate uptake increases substantially, contributing up to 60% of the heart’s oxidative substrate [6]. The occlusion of a coronary artery during a myocardial infarction significantly reduces the oxygen supply to cardiac muscle cells. This reduction decreases oxidative phosphorylation in mitochondria and increases the glycolytic rate in cardiomyocytes. High lactate levels are associated with shock, increased mortality, and a reduced response to percutaneous coronary intervention in patients with ST-elevation myocardial infarction [7, 8]. While elevated lactate levels over 24 h are associated with increased mortality, a study demonstrated that higher lactate clearance within the same timeframe correlates with reduced mortality in both men and women. The dynamic changes in lactate levels could, therefore, provide critical clinical insights, identifying patients with low lactate clearances who need intervention [7]. Lactate’s role extends far beyond just being a byproduct of anaerobic metabolism. A study revealed that lactate promotes the endothelial to mesenchymal transition via Snail1 lactylation, thereby exacerbating cardiac fibrosis and dysfunction following AMI [9]. Under physiological conditions lactate fulfils several other vital functions in the maintenance of homeostasis. These include the regulation of vascular smooth muscle cells, the regulation of hemodynamic and the electrophysiological activity of the heart [5]. Additionally, lactate protects cardiomyocytes from oxidative stress by neutralizing free radical species like superoxide anion (O2) and hydroxyl radical (OH). The effect depends on the prevailing lactate concentration [8, 10].

In this study, we found that a significant increase in lactate was accompanied by a simultaneous increase in hypoxanthine and glutamate levels. While metabolites of glutamate are associated with incident cardiovascular events [11], its structural homolog, glutamine is a cardioprotective agent in acute ischemic events [12, 13]. Hypoxanthine is an intermediate product of ATP degradation and a major contributor to oxidative stress and endothelial dysfunction. Importantly, hypoxanthine is an established key biomarker for myocardial ischemia [14]. Therefore, the simultaneous increase in lactate and hypoxanthine is interpreted as a cascade reaction leading to cellular energy failure and acute myocardial ischemia.

We have identified several other metabolites, which showed differences between CAD and AMI patients and may play a prognostic or diagnostic role for both groups. One of them, formic acid, also called methanoic acid, is a common organic acid used in a variety of industrial processes, including chemistry, agriculture and medicine. Exposure to formic acid has been associated with kidney damage in a number of species [15]. However, the effects of formic acid on the cardiovascular system are less studied. There is a paucity of research on the direct toxicity of formic acid and how it might affect the cardiovascular system. Nevertheless, a recent experimental study has demonstrated that formic acid reduces vascular tension and promotes vasodilatation [16]. The authors hypothesize that the vasodilation induced by formic acid is related to the endothelium via the NO/cGMP signaling pathway. This suggests that formic acid may positively influence the cardiovascular system by promoting vasodilation. Further studies are needed to explore the role of formic acid in atherosclerosis and vascular damage.

Another metabolite, phenylalanine is a neurotransmitter that is converted into dopamine, noradrenaline and serotonin in the brain. These neurotransmitters are crucial for the regulation of mood, sleep, emotional well-being and cognitive processes [17]. Moreover, phenylalanine serves as an energy source; especially when the body needs extra energy, it can be converted to glucose to meet these energy needs [18]. An essential function of phenylalanine stems in its antioxidant properties. It contributes to the formation of antioxidants that can protect the body from oxidative stress and damage caused by free radicals [19].

Phenylalanine, which increases with age, has emerged as a metabolite of interest due to its effects [20]. A study revealed that phenylalanine concentration has the strongest association with leukocyte telomere length [21]. This finding was significant in men but not in women [22]. These results align with another study demonstrating that phenylalanine levels are significantly elevated in older men but not in women [23].

Another metabolite that was significantly elevated in AMI patients was aspartic acid. These observations are consistent with the results of previously published studies [24, 25]. Aspartic acid, a non-essential amino acid, plays a crucial role in various metabolic pathways, including the malate-aspartate shuttle, which is vital for energy production in the heart. Alterations in aspartic acid levels can reflect disturbances in myocardial energy metabolism [25]. Aspartic acid is mostly synthesized by aspartate aminotransferase from oxaloacetate and glutamate, which are intermediates of the tricarboxylic acid cycle [26]. An increase in aspartic acid levels results in a direct concentration reduction of related substances within the TCA cycle [26]. This may lead to impairment or even interruption of the TCA cycle, which in turn causes a decrease in energy metabolism within the heart. In the context of advancing diagnostics and personalized medicine, the identification of novel metabolites for the early diagnosis of AMI is gaining increasing importance. With an AUC-value exceeding 0.8, aspartic acid in STEMI patients and in NSTEMI patients have emerged as potential biomarkers that warrant further investigation [25].

In this study, we used the KEGG metabolic pathway database to examine the composition and interrelationships of various metabolic pathways. KEGG was initiated in 1995 as a central database provided by the Japanese GenomeNet service [27] and was developed to facilitate the understanding of functional relationships at the cellular and organismal levels using genetic information. The method typically involves identifying a key metabolite and searching for its associated signaling pathways to better understand the interactions and collaborations of molecules within the body. The KEGG pathway analysis of our cohort (Table 3) revealed that one of the most affected KEGG pathways is the central carbon metabolism in cancer. This KEGG pathway is linked mainly to metabolic reprogramming of central carbon metabolism in cancer cells [28, 29]. Central carbon metabolism includes aerobic glycolysis, the pentose phosphate pathway in the cytoplasm and the TCA in the mitochondria. This pathway has been also identified and widely described in cardiomyocytes [30]. Cardiomyocytes are able to adapt to hypoxia or low oxygen by shifting nutrient uptake and utilization to maintain ATP provision and contractile function. A classic example of reprogrammed central carbon metabolism in cardiomyocytes is the shift in metabolism from oxidative phosphorylation to glycolysis at low oxygen concentrations and myocardial hypoxia/ischemia, which renders electron transfer inefficient and thus generates excess of reactive oxygen species [30].

According to our KEGG analysis, the alanine, aspartate, and glutamate metabolic pathway were also severely affected. Alanine, aspartate, and glutamate are amino acids that are derivatives and therefore play a crucial role in various metabolic pathways, including the synthesis of other amino acids, nucleotides, and energy production [31]. These derivatives are closely linked through transamination reactions, where amino groups are transferred between molecules. Glutamate acts as a central nitrogen donor, and alanine and aspartate are formed from glutamate through the transfer of amino groups [32]. In patients with cardiovascular disease, this pathway has been shown to be highly affected in patients with essential hypertension [33].

Another significant metabolic signature in our patients was found within the fructose and mannose metabolism KEGG pathway. Both fructose and mannose are hexose sugars, and their metabolic pathways lead to the production of glycolytic intermediates such as glyceraldehyde-3-phosphate and fructose-6-phosphate [34]. Fructose can enter glycolysis via fructose-1-phosphate, while mannose is converted into mannose-6-phosphate and then to fructose-6-phosphate [35]. These intermediates are metabolized by the same enzymes and pathways as glucose, contributing to energy production in cardiomyocytes [36]. It is therefore not surprising that changes in this pathway were observed in our cohort.

The butanoate metabolism comprised another pathway with alterations observed in our study. KEGG butanoate metabolism describes the metabolic fate of a series of short-chain fatty acids, that typically arise through intestinal digestion [37]. Among them, butyric acid or its conjugate base, butyrate, is used as an energy source not only in intestinal cells, but also in cardiomyocytes. It has been reported that in patients with heart failure, the microbiome, beeing responsible for butyrate production from dietary fibers, is inhibited, and the enzymatic apparatus for its metabolization is altered [38]. It is important to note that a recent study has proven that the short-chain fatty acid butyrate is an inotropic agent with vasodilatory [39] and cardioprotective properties [40].

## Conclusions

The metabolic differences identified in this study underscore the potential of metabolomics in combination with pathway analysis in distinguishing between CAD and AMI and highlight the metabolic dysregulation that occurs in these diseases. Our results suggest that these metabolites could serve as potential biomarkers, paving the way for personalized prognostic and diagnostic strategies.

## Limitations / Future direction

The present analysis was limited by the lack of an additional cohort for an external validation of the results. Hence, to determine whether biomarkers identified in the present study can effectively be incorporated into clinical practice for individualized prevention and diagnostic strategies, further large-scale studies are necessary to include longitudinal sampling to capture metabolic trajectories and demonstrate incremental diagnostic or prognostic value beyond established biomarkers and integrate metabolomic findings with clinical outcomes or imaging phenotypes.

## Supplementary Information

Below is the link to the electronic supplementary material.

Supplementary figure 3 (PNG 399 KB)

High Resolution Image(TIF 11.6 MB)

Supplementary figure 4 (PNG 504 KB)

High Resolution Image(TIF 7.66 MB)

## Data Availability

All data are available in the main text or the supplementary materials. The data that support the findings of this study are available from the corresponding author upon reasonable request.

## Abbreviations

ACS: acute coronary syndrome
AMI: acute myocardial infarction
CAD: coronary artery disease
NMR: nuclear magnetic resonance
